# Metabolism-Disrupting Chemicals Affecting the Liver: Screening, Testing, and Molecular Pathway Identification

**DOI:** 10.3390/ijms24032686

**Published:** 2023-01-31

**Authors:** Kristin Fritsche, Andrea Ziková-Kloas, Philip Marx-Stoelting, Albert Braeuning

**Affiliations:** 1 German Federal Institute for Risk Assessment, Department Food Safety, Max-Dohrn-Str. 8-10, 10589 Berlin, Germany; 2 German Federal Institute for Risk Assessment, Department Pesticides Safety, Max-Dohrn-Str. 8-10, 10589 Berlin, Germany

**Keywords:** endocrine-disrupting chemicals, metabolic disorders, testing, molecular pathways

## Abstract

The liver is the central metabolic organ of the body. The plethora of anabolic and catabolic pathways in the liver is tightly regulated by physiological signaling but may become imbalanced as a consequence of malnutrition or exposure to certain chemicals, so-called metabolic endocrine disrupters, or metabolism-disrupting chemicals (MDCs). Among different metabolism-related diseases, obesity and non-alcoholic fatty liver disease (NAFLD) constitute a growing health problem, which has been associated with a western lifestyle combining excessive caloric intake and reduced physical activity. In the past years, awareness of chemical exposure as an underlying cause of metabolic endocrine effects has continuously increased. Within this review, we have collected and summarized evidence that certain environmental MDCs are capable of contributing to metabolic diseases such as liver steatosis and cholestasis by different molecular mechanisms, thereby contributing to the metabolic syndrome. Despite the high relevance of metabolism-related diseases, standardized mechanistic assays for the identification and characterization of MDCs are missing. Therefore, the current state of candidate test systems to identify MDCs is presented, and their possible implementation into a testing strategy for MDCs is discussed.

## 1. Background

Metabolism-disrupting chemicals (MDCs) are environmental chemicals that can alter metabolic processes thereby influencing the onset of metabolic diseases such as obesity, type II diabetes (T2D), or fatty liver. Historically, such chemicals have been first termed obesogens by Grun and Blumberg and they further hypothesized that susceptibility to obesity starts during development and can be influenced by specific endocrine-disrupting chemicals (EDCs) [1,2]. With increasing evidence, the hypothesis has been expanded and the term MDCs has been defined [3,4]. MDCs affect multiple organs, including the thyroid, intestine, pancreas, stomach, and other organs, but the main targets of MDCs are the liver and adipose tissue [5]. Here, we focus on the liver as the target organ.

The liver is the central organ of glucose and fatty acid metabolism, reached by enterally absorbed nutrients and xenobiotics via the portal vein. Compounds and metabolites that have entered the body through other routes of exposure are transported to the liver via the hepatic artery. Branchings of the two blood vessels converge in the periportal areas of the so-called liver lobules, the functional units of the liver, from where blood flows through the sinusoids towards the central vein. Sinusoidal endothelial cells are specialized, fenestrated liver cells that allow contact between the blood and the hepatocytes surrounding the sinusoids. The space between sinusoids and hepatocytes, termed space of Disse, harbors two other cell types of the liver, namely Kupffer cells and Ito cells. Kupffer cells are the resident macrophages of the liver, whereas Ito cells are stellate cells, required for fat and vitamin A storage [6].

**Figure 1:**
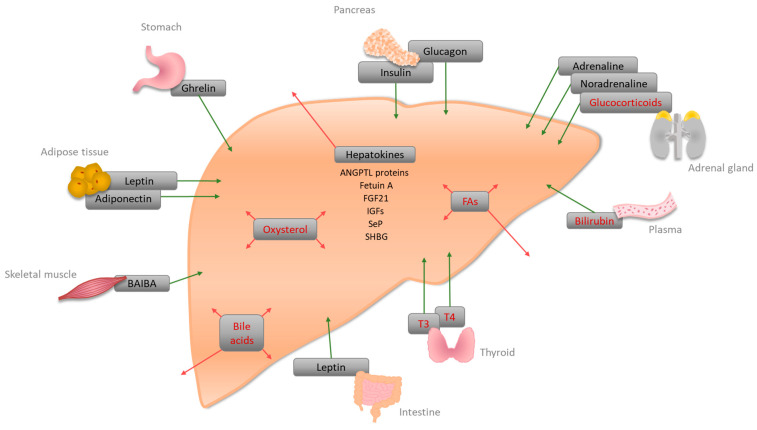
Schematic representation of liver-specific signaling molecules. The liver senses and response to hormones synthesized in the liver (red arrows) or other tissues (green arrows). Interactions of the displayed signaling molecules with other tissues than the liver are not shown. Signaling molecules that act via nuclear receptors in the liver are highlighted in red. BAIBA: β-aminoisobutyric acid; ANGPTL: Angiopoietin-like; FGF21: Fibroblast growth factor 21; IGFs: Insulin-like growth factors; SeP: Selenoprotein P; SHBG: Sex-hormone-binding globulin; T3: 3,5,3′-triiodothyronine; T4: Thyroxine; FAs: Fatty acids.

Most of the liver’s metabolic functions are executed by hepatocytes. They sense and respond to endocrine signals from adipose tissue, stomach, pancreas, intestine, thyroid, adrenal gland, skeletal muscle, and brain, as well as to liver-specific signaling molecules (Figure 1) [7]. In addition, the liver secretes so-called hepatokines, which have paracrine and endocrine effects on glucose and lipid metabolism [8].

Energy metabolism is among the most prominent functions of the liver. In the fed state, the liver responds to blood levels of glucose and insulin. Hepatic glucose is converted to glycogen (glycogenesis) for storage purposes and metabolized to pyruvate that either serves as an energy supply via mitochondrial oxidation or is used to synthesize fatty acids (FAs) through de novo lipogenesis [9]. FAs from the bloodstream or lipogenesis are esterified with glycerol-3-phosphate or cholesterol for the production of triacylglycerol (TAG) and cholesterol esters, respectively. These products are either stored as lipid droplets or released into the bloodstream as very low-density lipoproteins (VLDL). FAs also serve as precursors for phospholipids that are essential components for cell membranes, surface layers of lipid droplets, VLDL, and bile [10]. In the fasted state, or in response to stress, instead, glycogenolysis and gluconeogenesis lead to the production of glucose and its release into the bloodstream [11,12,13]. Additionally, lipolysis is induced in adipose tissue, which provides FAs that undergo β-oxidation in hepatic mitochondria to generate energy and ketone bodies. Ketone bodies and glucose are essential metabolites, which act as energy sources for extrahepatic tissues during starvation. The liver further synthesizes and catabolizes most plasma proteins, secretes bile acids built from cholesterol, and plays a major role in many more biotransformation processes including amino acid metabolism, the inactivation of steroid hormones [14], the conjugation and secretion of bilirubin [15], and the metabolism of drugs and xenobiotics [16].

## 2. Signaling Molecules and Receptors Regulating Hepatic Energy Metabolism

Individual signaling molecules trigger downstream signaling cascades by acting on specific receptors. Those molecules, including insulin, leptin, ghrelin, glucagon, and catecholamines, activate metabolic pathways in hepatocytes by acting on the cells via receptors located on the cell surface [17,18,19,20,21]. Metabolites, such as FAs, glucose, and amino acids, instead, mainly enter the cells using specific transporters [22,23,24], while others such as low-density lipoprotein (LDL)-cholesterol are absorbed by receptor-mediated endocytosis [25]. Other modulators of hepatic energy metabolism such as bile acids, FAs, thyroid hormones, and glucocorticoids enter the cells and deliver their signals by affecting the activity of certain nuclear receptors (NRs) (Table 1).

Hepatic NRs involved in energy metabolism and responses to xenobiotic exposure include the peroxisome proliferator-activated receptors (PPARs), PPARα, PPARβ/δ and PPARγ, the pregnane-X-receptor (PXR), the constitutive androstane receptor (CAR), the liver-X-receptor (LXR), the farnesoid-X-receptor (FXR), the thyroid receptors (TRs), as well as the vitamin D receptor (VDR). All of them may heterodimerize with the retinoid-X-receptor (RXR), which is activated by 9-cis retinoic acid [26,27,28]. PPARs are key players in lipid metabolism: In the fasted state, PPARα senses FAs and upregulates genes associated with lipid catabolism and ketone body synthesis [29]. PPARβ/δ, instead, increases the production of monounsaturated FAs, which are PPARα activators, and reduces the number of saturated FAs [30]. *PPARG* is only weakly expressed in the human liver, compared to adipose tissue [31], but its expression in hepatocytes increases in patients with non-alcoholic fatty liver disease (NAFLD), as well as in mice on a high-fat diet (HFD) [32]. Moreover, recent work in mice suggests that hepatic PPARγ is involved in FA uptake and diacylglycerol (DAG) synthesis [33]. Apart from lipid metabolism, PPARs are also involved in glucose metabolism and inflammatory processes [34]. The NRs CAR and PXR, as well as the aryl hydrocarbon receptor (AhR), are mostly known for their role in xenobiotic metabolism, but also play a role in lipid metabolism. Activation of PXR leads to the repression of genes coding for key enzymes in β-oxidation and ketogenesis [35]. CAR activation, instead, was shown to induce the anti-lipogenic protein insulin-induced gene 1 (*Insig1*) [36], and AhR activation transcriptionally regulates *Ppara* [37]. In addition, classic nuclear glucocorticoid receptors (GRs) in the liver are related to the fight-and-flight reaction by mediating the stress response [38].

Factors affecting liver metabolism via NRs cover hormones of various endocrine glands such as thyroid hormones and glucocorticoids, as well as metabolic compounds, i.e., bile metabolites and FAs. Thyroid hormone signaling in the liver is sensed mainly by the nuclear 3,5,3′-triiodothyronine (T3) receptor (TRβ) [39], and ligand binding leads to the release of co-repressors and the activation of TRβ target genes. Additionally, thyroid hormones also regulate other factors involved in lipid metabolism such as forkhead box protein O1 (FOXO1) activity [40]. Other external signaling molecules are glucocorticoids that bind to classic GRs [38] and affect gene expression via binding to glucocorticoid-responsive elements of the DNA leading to gluconeogenesis by lipolysis and ketogenesis [41]. Bilirubin, derived from the plasma, is conjugated and excreted to the bile, and reduces lipid accumulation by activation of PPARα [15]. NRs further play a major role in mediating the effect of liver-specific signaling molecules. FAs in the liver directly activate PPARs. PPARα signaling in the fasted state is crucial for hepatic lipid catabolism [42], and its liver-specific deletion causes steatosis in mice [43]. Activation of hepatic PPARα further increases energy production [44] and stimulates gluconeogenic genes [45], as well as the autophagy of lipids [46]. PPARδ/β activation instead reduces fasting glucose levels, while glycogen and lipid deposition, as well as de novo lipogenesis and glucose usage, are increased [30]. The role of PPARγ signaling in the liver is not yet fully understood, since both, the promotion and the prevention of hepatic steatosis have been observed for PPARγ agonists [47]. Hepatic oxysterols are intermediates in the bile acid synthesis pathway and are agonists for LXRα and LXRβ [48]. In response to elevated cholesterol, these NRs activate genes involved in cholesterol transport and catabolism [49]. Activation of LXR also induces *Srebp-1c*, thereby increasing de novo lipogenesis [50]. Bile acids are recognized by FXR, PXR, and VDR, which work in concert to regulate not only bile acid homeostasis and detoxification of xenobiotics, but also energy and glucose metabolism [51]. Primary bile acids activate FXR in the liver, whereas secondary bile acids activate PXR, VDR, CAR, and GPCRs, e.g., G protein-coupled bile acid receptor 1 (TGR5) in the gastrointestinal tract. Activation of FXR in hepatocytes decreases the levels of TAGs by induction of PPARα [52] and inhibits SREBP-1c- and LXR-mediated lipogenesis in mice [53]. Conversely, FXR was also shown to stimulate lipogenesis [54] and to increase body weight and glucose tolerance in mice under HFD [55]. The role of FXR in glucose metabolism is also contradictory: Activation of FXR has been shown both, to inhibit [56] and to stimulate phosphoenolpyruvate carboxykinase (PEPCK) [57]. Given the crosstalk of FXR signaling in other tissues, which has indirect effects on metabolic processes in the liver, FXR appears therefore to be an NR with very versatile functions [58].

The various peptidergic hormones acting via membrane-associated receptors affect liver metabolism through different downstream events in a complex modulatory manner. The main anabolic hormone of the body, insulin, is secreted by β-cells of the pancreatic islets of Langerhans and signals via the phosphatidylinositol-3-kinase (PI3K)/protein kinase B (PI3K/AKT) pathway in the liver. This leads to phosphorylation and inhibition of FOXO1, PPARγ coactivator 1-α (PGC-1α), and glycogen synthase kinase 3β (GSK3β), which inhibits gluconeogenesis in the fasted state [59]. Leptin signaling from adipose tissue, instead, occurs via the Janus kinase (JAK)/signal transducer and activator of transcription (STAT) pathway; the direct effect of this pathway on the liver is difficult to assess because it appears to crosstalk with insulin signaling [19]. While a complete loss of leptin causes impaired glucose homeostasis leading to obesity and a T2D phenotype [60], liver-specific leptin knockout in mice increases lipid accumulation in the liver but increases insulin sensitivity [61,62]. In the fasted state, pancreatic glucagon signaling in the liver acts antagonistically to insulin, activating genes involved in gluconeogenesis [18]. The underlying pathway is the G-protein-coupled receptor (GPCR)-mediated activation of cyclic adenosine monophosphate (cAMP)/protein kinase A (PKA) signaling cascade. PKA ultimately phosphorylates the transcription factor cAMP-response element binding protein (CREB), which induces the expression of gluconeogenic phosphoenol pyruvate carboxykinase (*Pepck*), glucose 6-phosphate catalytic subunit (*G6pase*), and pyruvate carboxylase [63]. Upon fasting, ghrelin is secreted by gastric cells and activation of the ghrelin receptor (GHSR1a) promotes lipogenesis via activation of the mammalian target of the rapamycin (mTOR)/PPARγ signaling pathway [64]. The catecholamines released by the adrenal medulla, adrenaline and noradrenaline, stimulate glycogenolysis via various adrenoreceptors belonging to alpha_1_-, alpha_2_-, or β-types, and each can be subdivided furthermore into three subtypes [65]. The effects of adrenergic receptors are transduced by G proteins involving for alpha_1_-adrenoreceptors G_q_-proteins (activating phospholipase C to stimulate inositol trisphosphate and DAG, leading to elevated calcium), for alpha_2_-adrenoreceptors G_i_-proteins (inactivating adenylate cyclase to decrease cAMP), and for β-adrenoreceptors by G_s_-proteins (stimulating adenylate cyclase resulting in increased cAMP levels). Several polymorphisms have been found concerning these adrenoreceptors, which may have clinical significance for the application of the numerous existing diverse pharmacological ligands [66]. Other external signaling molecules that are involved in metabolic processes in the liver are adipokines, e.g., adiponectin, suppressing gluconeogenesis [67] and acting via two membrane-associated adiponectin receptors [68], or myokines, e.g., β-aminoisobutyric acid (BAIBA), increasing β-oxidation in hepatocytes [69]. The multiple effects of BAIBA on metabolism and inflammation are mediated by the activation of AMP-activated protein kinase (AMPK) and involvement of regulators of gene expression, such as PPARα/δ/γ, PGC-1α, as well as transcription factors nuclear factor kappa B (Nf-κB) and sterol regulatory element-binding protein-1c (SREBP-1c) [70].

For crosstalking to other tissues, the liver secretes hepatokines, all of which affecting lipid and glucose metabolism [8]. Fetuin-A, a liver-secreted glycoprotein, is an inhibitor of insulin receptor tyrosine kinase [71]. High levels of hepatic glucose increase fetuin-A expression in liver cells possibly via extracellular signal-regulated kinase (ERK) 1/2 signaling [72]. Since circulating levels of fetuin-A are increased in obesity, metabolic syndrome, and T2D, and correlate to impaired insulin sensitivity and glucose intolerance [73], they represent a promising biomarker and might serve as a therapeutic target [74]. Similarly, also fibroblast growth factor 21 (FGF21) is of pharmacological interest since it causes weight loss, improved insulin sensitivity, and a decrease of triglycerides (TGs) and cholesterol levels in obese rodents [75]. FGF21 is induced in the liver by PPARα during fasting and refeeding and is secreted into the bloodstream leading to improved insulin sensitivity and glucose uptake [76,77]. FGF21 signals to adipose tissue (white and brown), the central nervous system (CNS), and the liver itself. FGF21 signaling in the hypothalamic-pituitary-adrenal (HPA) axis increases the release of glucocorticoids thereby increasing gluconeogenesis, ketogenesis, and β-oxidation in the liver [78]. It further enhances carbohydrate disposal and increases energy expenditure in the brown adipose tissue (BAT) of newborns [79]. Selenoprotein P (SeP) is a glycoprotein required for selenium homeostasis and its hepatic expression has been linked to insulin resistance [80]. Hepatic SeP expression is upregulated upon fasting, whereas insulin suppresses SeP expression through FOXO and PGC-1α [81]. It remains, however, to be determined whether higher SeP levels are a result or the cause of disturbed glucose metabolism [82]. Angiopoietin-like (ANGPTL) proteins, in addition to controlling angiogenesis, regulate also lipid metabolism, such as *Angptl3* and *Angptl4* [83,84]. The liver-specific *Angptl3* is induced by LXR activation [85], whereas PPARδ inhibits its expression [86]. Activation of ANGPTL6, also known as an angiopoietin-related growth factor, has been associated with several beneficial metabolic effects such as protection from steatosis, insulin resistance, and HFD-induced obesity [87]. Insulin-like growth factor-1 and 2 (IGF-1, IGF-2) is secreted by the liver and act in response to growth hormones released by the pituitary. IGF-1 treatment decreases blood glucose levels and improves insulin sensitivity [88], possibly by suppressing growth hormone secretion from the pituitary [89]. On the other hand, IGF-1 levels are reduced in NAFLD patients [90]. IGF-2 can be a key factor in steatosis initiation [91]. Similarly, the levels of the hepatokine sex-hormone-binding globulin (SHBG), binding specifically estrogens and androgens, are lower in patients with metabolic syndrome [92], and circulating levels of SHBG are considered to be a biomarker for insulin resistance T2D [93].

**Table 1:** Liver-specific signaling molecules. Hormones and metabolites acting via NRs (A) and NR-independent hormones (B) are listed.

Hormone	Site of Synthesis	Receptor(s)	Site of Action	Function
**A**				
Bile acid	Liver	FXR, PXR, VDR, GPCRs (TGR5, Sphingosine 1-phosphate receptor (S1P2))	Liver, intestine	Bile acid homeostasis, lipid, glucose, and energy homeostasis [51]
Bilirubin	Plasma	CAR, PXR, PPARα	Liver	Conjugation and secretion of bilirubin [94], an increase of FA oxidation, and decrease of lipid accumulation [15]
Fatty acids (FAs)	Liver, Adipose tissue	PPARα, PPARβ/δ, PPARγ	PPARα: liver, muscle, BAT, heart; PPARβ/δ: ubiquitous; PPARγ: adipose tissue, weak in Liver	PPARα: increase of fatty acid oxidation (FAO), a decrease of glucose uptake; PPARβ/δ: increase of FAO and glucose metabolism, decrease of inflammation [95]; PPARγ: might be involved in FA uptake and DAG synthesis [33]
Glucocorticoids (corticosterone, cortisol)	Adrenal cortex	GRs	Liver	Gluconeogenesis by lipolysis and ketogenesis [38,41]
Oxysterol	Liver	LXRα/LXRβ	Liver	Activation of LXRα, regulation of cholesterol metabolism [96]; LXR-induced *Srebp-1c* increases de novo lipogenesis [50]
Thyroid hormones (T3, T4)	Thyroid	TRα, TRβ	Liver, kidney, bone, heart	Cholesterol metabolism, stimulation of FAO, activation of de novo lipogenesis, and glucose homeostasis [97]
**B**				
Adiponectin (adipokine)	White adipose tissue (WAT)	Adiponectin receptor 1 and 2 (AdipoR1/2)	Liver, skeletal muscle, WAT	Suppression of glucose production in the liver via activation of AMPK [67]
Adrenaline, noradrenaline	Adrenal medulla	Adrenoreceptors alpha_1_, alpha_2_, and beta	Liver	Glycogenolysis, increase of blood glucose [65,66]
Angiopoietin-like proteins (ANGPTL3, ANGPTL 6) *	Liver	-	Plasma	Increase of plasma TG level in mice via lipoprotein lipase inhibition [84]; activation of *Angptl6* has been associated with protection from HFD-induced obesity, insulin resistance, and hepatic steatosis [87]
β-aminoisobutyric acid (BAIBA)	Skeletal muscle	AMPK, transcription factors	Liver, WAT, skeletal muscle	Improvement of hepatic lipid metabolism via PPAR-mediated β-oxidation [69,70]
Fetuin A (α2-HS-Glycoprotein) *	Liver	-	Plasma	Inhibition of insulin receptor tyrosine kinase [71]
Fibroblast growth factor 21 (FGF21) *	Liver	-	Plasma	Fasting-induced hormone enhancing insulin sensitivity, lowering body weight, and increasing gluconeogenesis [98]
Ghrelin	Stomach	Ghrelin receptor (GHSR1a)	Liver, Agouti-related protein (ARGP)/neuropeptide Y (NPY) neurons, adipocytes	Increase of triglycerides by induction of lipogenesis-related gene expression [64]
Glucagon	Pancreas	Glucagon receptor	Mainly liver, kidney	Gluconeogenesis [99]
Insulin	Pancreas	Insulin receptor	liver	Lipogenesis, cholesterol uptake, and synthesis [100]
Insulin-like growth factors-1 and -2 (IGFs) *	Liver	IGF receptors -1 and -2	Plasma	IGF-1 decreases blood glucose levels, and improves insulin sensitivity [88,89,90]. IGF-2 can be a key factor in steatosis initiation [91]
Leptin	Adipose tissue, small intestine	Leptin receptor	Liver, hypothalamus, and several other tissues	Lack of hepatic leptin leads to increased lipid accumulation in the liver [62]
Selenoprotein P (SeP) *	Liver	-	Plasma	Glycoprotein; hepatic expression has been linked to insulin resistance [80]
Sex-hormone-binding globulin (SHBG) *	Liver	SHBG-receptor	Plasma	Circulating levels of SHBG are a biomarker for insulin resistance and type II diabetes [93]

* hepatokines.

## 3. Examples of Compounds Affecting the Liver and Inducing Metabolic Changes

Several pathways, receptors, or signaling molecules can be affected by a variety of substances, resulting in metabolic changes (Table 2). Well-described endpoints of metabolic changes in the liver are the excessive accumulation of lipids in hepatocytes (steatosis) and the accumulation of bile (cholestasis), both being already summarized in constantly developing adverse outcome pathways (AOPs) [101,102,103]. Importantly, also serum glucose levels are indicative of metabolic changes in the liver since the disturbance in hepatic thyroid hormone signaling leads to altered hepatic glucose output contributing to the induction of insulin resistance [104,105]. Several substances have been shown to cause these effects in vivo and/or in vitro. However, understanding the underlying mechanisms is complicated not only because species-specific differences might exist, or because some substances crosstalk via multiple receptors, but also because some MDCs may cause metabolic changes that manifest later in life or in the next generation. Moreover, some of them require additional metabolism-impairing factors such as an HFD. A comprehensive review in this regard is given by Heindel et al. [3]. We here describe, in short, the known mechanisms of action of different chemical classes of MDCs, focusing in each case on the best-characterized class-specific substance.

### 3.1. Bisphenols

Bisphenol A (BPA), a plasticizer used for the production of different plastics and resins, is being discussed controversially due to its estrogenic activity. It affects hepatic metabolism even at low concentrations [106]. Multiple mechanisms have been shown by which BPA induces hepatic lipid accumulation. In vitro, mitochondrial dysfunction was observed in the liver cell line HepG2 [107], while in vivo mouse studies showed the upregulation of genes involved in lipogenesis [108] and the dysregulation of autophagy [109], as well as an increase in *Pparg* expression upon in utero exposure [110]. Perinatal exposure of rats was further shown to affect hepatic glucose homeostasis possibly by epigenetic reprogramming in early development [111]. Interestingly, other bisphenols such as bisphenol S (BPS), bisphenol F (BPF), and bisphenol AF (BPAF) show different effects on lipid and glucose metabolism. Especially BPAF was shown to decrease free FAs and TGs in the liver, whereas BPS acted similarly to BPA, and BPF showed only a minor influence on hepatic lipid content. BPS and BPAF further heavily interfered with glucose metabolism increasing glucose and glycogen contents in mouse liver [112].

### 3.2. (Tri-)azoles

Substances from the class of azole antifungals target the fungal cytochrome P450 enzyme CYP51, thereby inhibiting cytochrome P450 catalytic activity [113]. Azole compounds are widely used agrochemical fungicides, but also for the therapy of fungal infections in humans. Side effects observed in mammals, apart from steatotic effects, are severe toxicity and disturbance of steroid hormone synthesis [114,115]. Recent in vivo and in vitro studies show that steatotic effects are mediated by the NRs CAR and PXR [116,117]. These NRs are, amongst others, defined as molecular initiating events in the AOP for liver steatosis [102]. However, some substances do not induce key genes/proteins according to the AOP, indicating a need for further improvement of the AOP [118]. Activation of other NRs by azoles leading to adverse outcomes apart from steatosis is reviewed in detail by Marx-Stoelting et al. [119].

### 3.3. Polyfluoroalkyl Substances (PFAS)

PFAS comprise a large group of compounds containing at least one perfluoroalkyl moiety. PFAS are industrial chemicals used, e.g., for the manufacturing of dirt-repellent surfaces or in firefighting foams, and some PFAS are ubiquitously present in the environment due to their stability and long half-life. PFAS have been associated with a variety of harmful health effects, such as cancer, immune system dysfunction, developmental and reproductive toxicity, as well as liver damage and hormone disruption [120]. The most investigated PFAS are perfluorooctane sulfonic acid (PFOS) and perfluorooctanoic acid (PFOA). Both of them are classified as persistent organic pollutants (POPs), and their use in the European Union has been restricted since 2009 and 2020, respectively [121,122]. Several derivatives of PFOA and PFOS are now being produced as substitutes, but studies regarding their potential adverse effects are still underrepresented [123,124]. The adverse effect steatosis of PFOS and PFOA, instead, has been shown in several in vitro and in vivo studies, and a putative mechanism is the inhibition of mitochondrial fatty acid β-oxidation in mouse liver [125]. Though several PFAS induce an increased expression of genes involved in FA and TG synthesis, only PFOS has been shown to act via PPARα in vivo using a *Ppara*-null mouse model [126]. Activation of other NRs has been observed in vivo, but neither PFOS nor PFOA activates human NRs apart from PPARα in vitro as shown by reporter gene assays in the human embryonic kidney cell line HEK293T [127]. Epidemiologically, PFOS and PFOA also correlate to increased serum total cholesterol levels and in some cases to TG levels. In rodents, however, PFOS induced lower serum cholesterol and increased liver fat accumulation [128], and in vitro in human HepaRG liver cells, genes of the cholesterol biosynthesis pathway have been found repressed by PFOA, PFOS, and perfluorononanoic acid (PFNA), while TG levels were increased [129]. It remains to be determined whether mechanisms such as decreased liver-specific uptake of cholesterol lead to increased serum cholesterol levels, as speculated by Louisse et al. (2020). In addition to decreased hepatic TG levels, Behr and colleagues further observed decreased bile acid synthesis and dilatation of bile canaliculi in PFOS- and PFOA-treated HepaRG cells, both being indicators of cholestasis [130]. Ultimately, PFOA exposure also affects glucose homeostasis and increases insulin sensitivity in mice by modulating the PI3K/AKT signaling pathway [131].

### 3.4. Polychlorinated Biphenyls (PCBs)

PCBs, a group of persistent organic pollutants, have been industrially used for several applications, e.g., as insulators or as coolants. PCBs comprise a large group of substances that are classified as coplanar and non-coplanar based on the chlorine substitution of the two phenyl rings [132]. Coplanar PCBs are AhR agonists and therefore termed “dioxin-like” [133], whereas some non-coplanar PCBs (PCB 153 and 196) possibly act via PXR and CAR, therefore referred to as “phenobarbital-like” [134,135]. Both, coplanar and non-coplanar PCBs have been shown to increase TGs and free fatty acids (FFAs) leading to steatosis [136,137,138,139]). Interestingly, PCB 153 was further described as a diet-dependent MDC, leading to steatosis in mice upon HFD. When occurring in a mixture (Aroclor 1260), however, this effect was not observed [140].

### 3.5. Phthalates

Phthalates are industrial chemicals mainly used as plasticizing additives in various plastics, especially polyvinyl chloride. Harmful effects of phthalates on the liver have been known for decades [141] and the role of certain phthalates such as diethylhexyl phthalate (DEHP) or its metabolite mono-ethylhexyl phthalate (MEHP) in affecting liver lipid metabolism is well described. DEHP exposure leads to lipid accumulation and oxidative stress via activation of PPARα and the SREBP-1c signaling pathway in human HepG2 liver cells [142]. Further, DEHP activates human CAR2 leading to the induction of cytochrome P450 Family 2 Subfamily B Member 6 (CYP2B6) and CYP3A4 [143]. In vivo studies, instead, showed a lean phenotype and protection from diet-induced obesity upon DEHP treatment. Conversely, using a humanized PPARα mouse, the opposite was observed pointing out crucial inter-species differences [144].

### 3.6. Dioxins

Dioxins are highly persistent organic pollutants resulting for example from combustion processes. They are, as well as dioxin-like compounds described above, AhR agonists, and prolonged high-dose exposure lead to AhR-mediated multi-organ toxicity, wasting syndrome, and death [145]. Low-dose exposure to 2,3,7,8-tetrachlorodibenzo-*p*-dioxin (TCDD) leads to an AhR-induced gene expression profile resembling insulin resistance [146]. TCDD exposure further leads to the inhibition of VLDL-TG secretion, possibly contributing to the steatotic effect of TCDD [147].

### 3.7. Alkylphenols

4-Nonylphenol (4-NP), used for example in the synthesis of plastics and resins, belongs to the persistent EDCs and causes estrogenic effects affecting the development of the reproductive system [148,149]. It accumulates in the liver and was shown to induce hepatic steatosis [150] and NAFLD [151]. It is, however, still unclear, how 4-NP induces lipid accumulation in the liver. Gene expression data from the aforementioned study by Kourouma et al. (2015) indicated processes of extrinsic apoptosis and insulin resistance in mouse liver. Studies investigating the mechanisms by which 4-NP induces steatosis, especially in human cells, are still missing.

### 3.8. Organotins

Organotins, industrial chemicals used, amongst others, as plastics additives and biocidal compounds, are well-described MDCs causing adipogenesis and associated metabolic disturbances even transgenerationally [152]. Prenatal tributyltin (TBT) exposure causes lipid accumulation in the liver of the F1, F2, and F3 generations accompanied by increased expression of genes involved in lipogenesis, FA synthesis, glycerol uptake, and lipolysis (*Ppara*, *Pparg*, *Srebp-1c*, fatty acid synthase (*Fasn*), glycerol kinase (*GyK*), acyl-CoA oxidase (*Acox*) [153]. Moreover, Zuo et al. observed, apart from hepatic steatosis, also the reduction of hepatic resistin and adiponectin, resulting in hyperinsulinemia and -leptinemia in mice [154]. TBT can activate PPARγ/RXR in transformed green monkey kidney fibroblast cells (Cos7 cells) [155], and TBT chloride also activates LXRα/RXR and, weakly, PPARα/RXR in Cos1 cells [156]. Interestingly, studies in zebrafish detected sex-specific differences in the lipogenesis-specific gene expression response of the zebrafish liver upon TBT exposure [157]. Surprisingly few studies investigated organotin-mediated adverse effects in human liver cells in detail. Stossi and others confirmed the induction of lipogenesis via PPARγ/RXRα in vitro in HepaRG human liver cells by TBT [158]. On the other hand, Qiao and colleagues found that dibutyltin dilaurate (DBTD) treatment of human HL7702 liver cells decreased TG content dose-dependently possibly via suppression of the mTOR pathway [159].

### 3.9. Polycyclic Aromatic Hydrocarbons (PAHs)

PAHs such as benzo[a]pyrene (BaP) result from incomplete combustion of organic material. PAHs are mostly activators of AhR and some compounds from this group are known for their mutagenic and carcinogenic potential [160]. Affecting liver lipid metabolism, however, it appears that additional factors are required. Bucher et al. found that the progression of steatosis in vitro and in vivo is induced upon co-exposure to BaP and ethanol using the human HepaRG liver cell line, as well as a hybrid human fibroblast-rat liver cell line as in vitro model, and obese zebrafish as in vivo model [161]. They further found that mitochondrial dysfunction, increased reactive oxygen species (ROS), apoptosis, and necrosis might be involved and possibly dependent on AhR activation [162]. Ortiz et al. instead observed that prenatal exposure to BaP induces hepatic lipid accumulation and regulation of genes involved in FA β-oxidation in female offspring. Interestingly, mice deficient in glutathione synthesis did not exhibit this phenotype, and the resistance to BaP was associated with hepatic downregulation of genes involved in lipid biosynthesis and upregulation of antioxidant genes [163]. Recent work using mixtures of PAHs and HepaRG cells revealed that while BaP activates AhR, the non-carcinogenic pyrene and fluoranthene activate CAR instead. However, when occurring in a mixture, the transactivation of CAR is reduced affecting also the induced *CYP2B6* expression [164].

### 3.10. Non-Steroidal Estrogens

Non-steroidal estrogens, such as diethylstilbestrol (DES), which has been pharmacologically used as an estrogen analog in past years, are agonists of the estrogen receptors ERα and ERβ and are suggested to be MDCs [165,166]. Perinatal exposure to DES induces the expression of *Pparg* and its target genes in adipocytes leading to increased body and liver weight [167]. In the mouse liver, neonatal exposure to DES alters bile acid and TG metabolism, mediated by the small heterodimer partner (SHP), which is maintained in adulthood possibly by epigenetic processes [168]. Further, in rats treated with DES, apolipoprotein E (ApoE) secretion is suppressed leading to disruption of steroidogenesis in adrenal glands [169].

### 3.11. Organochlorines

Vinyl chloride, the basic module of the plastic material polyvinyl chloride, is well known for its multiple adverse effects on the liver steatosis, fibrosis, and hepatocellular carcinoma [170,171]. It is, however, considered safe at lower concentrations [172], although it was shown to enhance TG accumulation in mice with HFD-induced steatosis [173]. Further, it might be a “second hit” environmental factor for the progression of HFD and metabolic syndrome causing oxidative and endoplasmatic reticulum stress by impairment of aldehyde dehydrogenase 2 family member (ALDH2) function in mice [174,175].

### 3.12. Organophosphates

Organophosphates are frequently used active compounds in pesticides or as flame retardants and some of them are accepted endocrine disruptors that show affinity to several nuclear receptors such as ERα/β, PXR, androgen receptor (AR), or GR [176]. Recent studies revealed that organophosphorus flame-retardants (OPFRs) cause lipid accumulation in human hepatocellular HepG2 cells. However, different classes of tested OPFRs show activation of different pathways leading to lipid accumulation. Halogenated OPFRs (tris(2-chloroethyl)phosphate (TCEP), tris(2,3-dibromopropyl) phosphate (TBPP), tris(2-chloro-1-(chloromethyl)ethyl) phosphate (TDCPP), and tris(2-chloroisopropyl)phosphate (TCPP)) cause TG accumulation via de novo FA synthesis and inhibition of β-oxidation, and aryl-OPFRs (triphenyl phosphate (TPhP) and tricresyl phosphate (TCP)) additionally induce total cholesterol deposition through PPARγ and SREBP2 signaling in HepG2 cells. Mitochondrial dysfunction was observed upon treatment with all substances [177]. Negi et al. (2021) showed that several novel flame retardants (e.g., tricresyl phosphates (TMPP, TPhP), 2-ethylhexyl diphenyl phosphate (EHDPP) and tris (1,3-dichloropropyl )phosphate (TDCIPP)) could induce lipid accumulation and enhance hepatic steatosis via PPARγ activation and increased de novo FA synthesis pathway [178]. The insecticide malathion, instead, promotes insulin resistance, inflammation, and steatosis in rats possibly via oxidative stress [179]. Moreover, it stimulates glucose release into the blood via increased hepatic PEPCK and glycogen phosphorylase activity [180].

### 3.13. Heavy Metals

Heavy metals, such as cadmium or arsenic, rank among the most hazardous EDCs, and exposure occurs largely via food intake and drinking water, respectively, but also via cigarette smoke and dermal contact with certain cosmetic products [181]. The effect of cadmium on carbohydrate metabolism has been shown already in 1974 when exposure of rats to cadmium chloride was shown to lead to increased gluconeogenesis and decreased hepatic glycogen [182]. Cadmium exposure further affects lipid metabolism; however, differences between higher and lower doses have been observed. Using an in vivo rat model and 10 weeks exposure, Alshehri et al. observed the induction of NAFLD by upregulation of SREBP1/2 and downregulation of PPARα and associated these effects with suppression of the Sirtuin 1 (SIRT1)/FXR axis [183]. Young and others, instead, did not observe the development of NAFLD in rats with exposure to cadmium in utero that continued throughout their whole life. They found, however, that HDF-induced steatosis was exacerbated at higher doses and, conversely, attenuated using a low-dose treatment [184].

**Table 2:** Selected compounds affecting the liver and inducing metabolic changes.

Substance	Putative Mechanism	Effect	Test System	Reference
Bisphenols (BPA)	ROS production	Lipid accumulation	In vitro (HepG2, 72 h)	[107]
	Upregulation of genes involved in lipogenesis	Accumulation of liver TGs	In vivo (mice, 28 days)	[108]
	Inhibition of autophagy possibly via mTOR	Hepatic lipid accumulation	In vivo (male mice, 8 weeks) and in vitro (HepG2, primary hepatocytes)	[109]
	Upregulation of *Pparγ*	Increase of hepatic triglycerides	In vivo (in utero exposure of male mice, days 9 to 16 of pregnancy)	[110]
	Promoter methylation of hepatic glucokinase	Increase in hepatic glycogen content	In vivo (rats, throughout gestation and lactation)	[111]
(Tri-)azoles (propiconazole, tebuconazole)	Activation of PXR, CAR, regulation of steatosis-related genes	Triglyceride accumulation	In vitro (HepG2/HepaRG, 24 h)	[117]
PFAS (PFOS, PFOA)	PPARα	Increase in liver weight and cell size, increased lipid accumulation, liver steatosis	In vivo (mice, 7 days)	[126]
	Inhibition of mitochondrial FA β-oxidation	Hepatic steatosis	In vivo (mice, up to 21 days)	[125]
	Decrease of CYP7A1	Decreased levels of bile acids	In vitro (HepaRG, 24 and 48 h)	[130]
	Modulation of PI3K-AKT pathway	Altered glucose homeostasis and induction of insulin sensitivity	In vivo (mice, 28 days)	[109]
PCBs (PCB 126, Aroclor 1260)	PCB126: Increased expression of *Nr1i3* (*Car)*, induction of *Cyp1a2*, *Cyp2b10*, and genes involved in lipid metabolism	Increased TGs and free FAs leading to steatosis	In vivo (male mice, 2 weeks)	[137]
	Aroclor 1260: PXR, CAR, AhR (agonistically) PPARα (antagonistically)	Induction of *CYP1A1*, *CD36* (AhR), induction of *CYP3A4* (PXR)	In vitro (HepG2 and primary human hepatocytes, 24 h)	[137]
Phthalates (DEHP, DBP, MEHP)	Activation of SREBP-1c and PPARα	Lipid accumulation	In vitro (HepG2, 48 h)	[142]
	Activation of CAR2, induction of *CYP2B6* and *CYP3A4*	-	In vitro (HepG2, 48 h)	[143]
Dioxin (TCDD)	AhR	Insulin resistance-like phenotype	In vivo (mice, 18 days)	[146]
		Inhibition of VLDL-TG secretion	In vivo (mice, 7 days)	[147]
Alkylphenols (4-NP)	Contributing factors: Fas Cell Surface Death Receptor (FAS)/FAS ligand (FASL), Tumor Necrosis Factor alpha (TNFα), Caspase-9 mRNA activation	Hepatic steatosis and apoptosis	In vivo (male rats, 30 days)	[150]
	-	Steatosis and NAFLD	In vivo (male rats, 90 days chronic exposure)	[151]
	Increased activity of hexokinase and phosphofructokinase, a decrease of glycogen phosphorylase, increased H_2_O_2_ generation and lipid peroxidation, decreased protein level of insulin receptor (IR), IR substrate (IRS)-1 and IRS2 and PI3K	Short-term: impaired liver glucose homeostasis	In vivo (rats, 7 days)	[174]
		Long-term: downregulation of insulin signaling	In vivo (rats, 45 days)	[173]
Organotins (TBT)	Activation of PPARγ and RXR, increased gene expression of genes involved in lipogenesis, FA synthesis, glycerol uptake, lipolysis	Hepatic lipid accumulation	In vivo (adult mice upon in-utero exposure throughout pregnancy)	[153]
	PPARγ/RXR-induced induction of lipogenesis	Increased hepatic TGs, steatosis	In vivo (mice upon in utero exposure from E12–18) and in vitro (HepaRG, 14 days)	[155,158]
	Reduction of hepatic resistin and adiponectin, an increase of plasma resistin and leptin	Hepatic steatosis, hyperinsulinemia, and hyperleptinemia	In vivo (male mice, 45 days)	[154,185]
	Dose- and sex-specific alterations of genes involved in lipogenesis	Accumulation of hepatic triglycerides in males, hepatomegaly in females	In vivo (zebrafish, pre-hatch-9 months)	[157]
PAHs (BaP, fluoranthene)	AhR (some BaP metabolites [186]), gene expression related to FA β-oxidation	Hepatic steatosis	In vivo (mice upon in utero exposure from gestational days 7–16)	[163]
	CAR (pyrene and fluoranthene) and CYP2B6 induction		In vitro (HepG2 and HepaRG, 24 h)	[164]
Non-steroidal estrogens (DES)	ERα, SHP	Increases liver weight, alteration in bile acid and triglyceride homeostasis	In vivo (mice: neonatal exposure, 5 days)	[167,168]
	Suppression of ApoE secretion → reduction of serum High-Density-Lipoprotein (HDL)/cholesterol levels	Steroidogenesis disruption in adrenal glands	In vivo (male rats, 24 h)	[169]
Organochlorines (Vinyl chloride)	Decreased mitochondrial respiration, endoplasmatic reticulum stress, impaired ALDH2 function	Enhanced TG accumulation in HFD-induced hepatic steatosis	In vivo (mice, 12 weeks)	[173,174]
	Increase of FA synthesis, possibly via endoplasmatic reticulum- and oxidative stress	Hepatic steatosis	In vivo (mice: sub-chronic exposure, 16 weeks)	[175]
Organophosphates (OPFRs, Malathion)	ERα/β, PXR, AR, GR	-	-	[176]
	*De novo* FA synthesis, inhibition of β-oxidation, induction of total cholesterol deposition, mitochondrial dysfunction	Lipid accumulation	In vitro (HepG2, 24 h)	[177,178]
	Oxidative stress	Promotion of insulin resistance, hepatic steatosis	In vivo (rats, 28 days)	[179]
	Increased hepatic PEPCK and glycogen phosphorylase activity	Increased glucose release into the blood	In vivo (rats, sub-chronic exposure, 4 weeks)	[180]
Heavy metals (Cadmium, cadmium chloride)	Increased activity of key enzymes involved in glucose production	Increased gluconeogenesis	In vivo (rats, 45 days)	[182]
	HFD-related altered levels of metallothionein	Exacerbated (higher-dose exposure) and attenuated (low-dose exposure) HFD-induced steatosis	In vivo (mice, whole life exposure, starting in utero)	[184]
	Upregulation of SREBP1/2 and downregulation of PPARα, suppression of SIRT1/FXR axis	Induction of NAFLD	In vivo (rats, 10 weeks)	[183]
	Differential expression of NAFLD-associated genes	Increased liver lipids	In vivo (male mice, low-dose exposure)	[187]
		Accumulation of TG, upregulation of steatotic marker genes	In vitro (HepaRG and HepG2 cells)	[188]

## 4. Testing Methods for MDC Identification

There is only one guidance document for regulatory authorities on evaluating chemicals for endocrine-disrupting properties available so far, written by the European Food Safety Authority (EFSA) and the European Chemicals Agency (ECHA), and supported by the Joint Research Center (JRC) (Table 3) [189]. It is, however, merely limited to estrogen-, androgen- and thyroid-mediated endocrine disruption, and chemicals that interfere with steroidogenesis. Therefore, there is an urgent need to address further endocrine systems/pathways, e.g., for metabolic disorders, by the development of new guidance documents. Up to now, hepatic effects of chemicals are classically studied within the course of repeated-dose animal trials mostly conducted for 28 or 90 days, preferentially using rodent species [190,191]. Measured endpoints comprise organ weight, organ-to-body weight ratios, and especially a detailed histopathological examination of tissue samples. Indirect evidence for adverse effects on the liver can be further contributed by clinical chemistry data, indicating e.g., hepatic cell death by elevated serum levels of hepatocellular enzymes. Additionally, histopathology data is used to provide information about a plethora of hepatic manifestations of toxicity. Moreover, also adaptive responses such as hepatocellular hypertrophy can be recorded, as is often observed following exposure to agonists of some NRs. Histopathology is also able to deliver information regarding metabolic alterations; identifying, e.g., hepatocellular fat vacuoles pointing towards an imbalance of fatty acid metabolism, the proliferation of fatty acid-metabolizing peroxisomes, or clear cell changes indicating accumulation of glycogen [192]. While histopathological examination of tissue slices has proven very valuable for assessing the hepatotoxicity of compounds, the endpoints classically used for interpretation of the results from regulatory studies are mostly limited with respect to their ability to identify molecular mechanisms and/or targets. Furthermore, the long duration and ethical constraints make such studies not ideal for large-scale screening approaches. Potential interactions of the test compounds with specific dietary factors, or the effect of a test compound on the organism’s ability to face metabolic/nutritional challenges, are also not addressed. Another drawback is pronounced differences between rodent and human liver cells in their response to some NR agonists, e.g., CAR and PPARα, thus impeding proper interpretation of study results [193,194,195]. Histopathological data is also sometimes more difficult to quantify, as compared to results obtained by bioanalytical methods.

In order to refine animal testing, different strategies can be followed. First, additional techniques may be employed to increase the information yielded from tissue samples after a study has been conducted following the established guidelines. With respect to metabolic alterations, the potential number of metabolic intermediates to be measured appears almost infinite and a complete overview of this topic is far beyond the scope of this work. Such metabolic analyses may use different experimental platforms and include targeted strategies to measure a single metabolite or a panel of pre-defined metabolites. Exemplarily, gas chromatography-based analysis of triglyceride levels in liver tissue is listed as a targeted method that is suited to gain quantitative information about metabolites highly relevant in hepatic steatosis [196]. Another option could be the use of non-targeted techniques [197]. Metabolomics analyses have clearly shown their scientific value in basic research, while problems with standardization and validation so far preclude their routine use in regulatory testing [198]. A second, different strategy could be followed by amending the standard repeated-dose study protocols with additional tests aimed at investigating the ability of the organism to cope with nutrition-related challenges. With respect to this field, glucose and insulin tolerance tests are given as examples [199,200]. While being routinely used in basic science, formal validation and standardization of such approaches are still pending.

In vitro studies with human cell cultures are an alternative to animal testing, avoiding ethical issues and providing opportunities for high-throughput testing yielding mechanistic information without the problem of inter-species differences. A plethora of human liver cell lines are available, and numerous biochemical assays exist which are capable of recording changes in enzyme activities, metabolites, transcription factor activation, the activity of signaling cascades, or the expression of genes and proteins. Knowledge about the molecular mechanisms leading to a certain adverse outcome is a prerequisite to establishing a meaningful mechanism-based testing strategy. In this regard, the AOP concept is a sequential chain/network of causatively linked key events (KE) at different levels of biological organization, connecting a molecular initiating event (MIE) to an adverse outcome [101,102]. AOPs can be used as a basis for risk assessment. With respect to metabolic alterations in the liver, only a few AOPs have been established so far [201]. Using information from the AOP for liver steatosis, recent work has established an in vitro test battery aiming at identifying the steatotic potential of chemicals in human liver cells [118,202]. Assays used in that toolbox comprise reporter gene analyses of NR activation as the MIE of hepatic steatosis, measurement of alterations in mRNA and protein expression, mitochondrial parameters, and different techniques to monitor triglyceride accumulation, thus covering the essential elements as proposed in the liver steatosis AOP [101,102,178]. However, extensive validation and standardization efforts need to be undertaken prior to the use of such in vitro test batteries in regulatory settings, and also the definition of adversity in in vitro studies poses an obstacle for regulatory use. Nonetheless, these test methods are already useful with regard to screening purposes for prioritization of further testing, as well as for obtaining mechanistic information about the mode of action of a test compound. As an additional in vitro approach going beyond the existing AOP, Lichtenstein et al. have identified a transcript marker panel to predict triglyceride accumulation in vitro [203]. Using that type of approach, different marker sets for specific metabolic endocrine effects of chemicals might be defined in the future, allowing for the identification of different types of metabolic endocrine disrupters within one transcriptomic analysis.

In order to replace, refine and reduce (3Rs) animal testing, the ECHA developed a read-across assessment framework (RAAF) to make use of relevant information from tested substances to predict the properties of the target [204]. This approach is used within the REACH regulation and enables to close data gaps.

**Table 3:** Validated or suggested test approaches.

Method	Principle	Effects Analyzed	Status	Reference
OECD standardized test guidelines for evaluating EDs	Repeated-dose 28-day/90-day study	Body and organ weight, (histo)pathology, clinical chemistry	Harmonized test guidelines approved for regulatory use	[190,191]
In vivo endpoints (to characterize metabolic phenotype)	Glucose and insulin tolerance test (GTT, ITT)	Blood glucose levels are measured upon administration of glucose/insulin	Additional techniques might be added as new endpoints	[199,200]
	Non-targeted metabolomics	Non-targeted liquid chromatography/mass spectrometry (LC/MS)		[196,197,205,206]
	Targeted metabolomics	Triglyceride measurement by gas chromatography		
In silico approach	Computerized models (e.g., (Q)SAR) predicting physicochemical, biological, and environmental fate properties based on chemical structure	Interaction of a chemical with a defined biological target (modeling of molecular docking simulations to receptors)	Use for identification of MIEs of AOPs	[178]
Grouping of substances and read-across	Use of relevant information from tested substances to predict the properties of target substances	Alternative approach for filling data gaps	In registrations submitted under the REACH regulation	[204]
In vitro toolbox	AOP-based in vitro assays measuring MIEs or KEs	Combinations of NR activation, gene and protein expression, lipid accumulation, mitochondrial respiration/dysfunction, formation of fatty liver cells	Use for AOPs	[118,178,202]
Transcriptomic signatures	In vitro model	Gene expression markers for accumulation of triglycerides		[203]

## 5. Conclusions

There is accumulating evidence that some chemicals contribute to metabolic disorders, both in laboratory animals and in humans. The current, mainly animal experiment-based testing strategies and guidelines applied in different fields of regulation, however, do not cover metabolism-related endpoints very well, thus posing a need for the development and validation of further testing methods for MDC assessment. This can be achieved based on increased knowledge of molecular mechanisms of toxicity, put together in AOPs as a basis for the development of test systems for individual modes of action, and subsequent implementation of in vitro tests as a testing battery for metabolic endocrine disruption. This will help to further improve risk assessment and consumer safety, along with the possibility to reduce the use of animals in toxicological testing.

## Data Availability

Not applicable.
